# Correction: Shu et al. Relationship between Circulating 25-Hydroxyvitamin D and Metabolic Syndrome in Chinese Adults: A Large Nationwide Longitudinal Study. *Nutrients* 2024, *16*, 1480

**DOI:** 10.3390/nu17101728

**Published:** 2025-05-20

**Authors:** Mi Shu, Yue Xi, Jie Wu, Lai-Bao Zhuo, Yan Yan, Yi-Duo Yang, Yue-Yue Feng, Hua-Qiao Tan, Hui-Fang Yang, Yu-Ming Chen

**Affiliations:** 1 Department of Epidemiology, School of Public Health, Sun Yat-sen University, Guangzhou 510080, China; shum7@mail2.sysu.edu.cn (M.S.); xiyue5@mail2.sysu.edu.cn (Y.X.); zhuolb@mail2.sysu.edu.cn (L.-B.Z.); yany36@mail2.sysu.edu.cn (Y.Y.); tanhq7@mail2.sysu.edu.cn (H.-Q.T.); 2 Yibicom Health Management Center, Guangzhou 510530, China; wujie6370@cvte.com (J.W.); yangyiduo@cvte.com (Y.-D.Y.); fengyueyue@cvte.com (Y.-Y.F.)

## Text Correction

The following two errors were identified in the main text [1]:

In the Results section (3.1. Participant Characteristics, fifth sentence): A factual error was found regarding the prevalence of Vitamin D (VD) deficiency. The original statement incorrectly indicated that “The VD deficiency prevalence was highest in summer and fall and decreased with age, and was most prevalent in the 18–44 age group”. This has been corrected to state that “The VD deficiency was actually lowest in summer and fall, and while it did decrease with age, it remained most prevalent in the 18–44 age group”.
In the Discussion section (second sentence): There was an error due to a misalignment of data in Table 1. The initial version incorrectly reported the prevalence of Metabolic Syndrome (MetS) as 26.9%. This has been amended to the correct prevalence of 27.5%.

## Errors in Tables

In the original publication, the following mistakes were also present in Tables 1 and S1:

In Table 1, the outcomes for “High Triglycerides, Low HDL-C, Hypertension, and Hyperglycemia” were not correctly aligned with their respective variables.

In Table S1, data related to “Age: 18–44, 45–59; Metabolic Syndrome” was misaligned.

The corrected version of Table 1 and Table S1 are provided below.

**Table 1:** Baseline characteristics of study participants based on quartiles (Q) of serum 25(OH)D levels.

Variables	Overall	Q1	Q2	Q3	Q4	*p*-Value
*n*	23,810	6663	6172	5865	5110	
Sex, *n* (%)						0.490
Male	12,596 (52.9)	3539 (53.1)	3246 (52.6)	3068 (52.3)	2743 (53.7)	
Female	11,214 (47.1)	3124 (46.9)	2926 (47.4)	2797 (47.7)	2367 (46.3)	
Age, y	43.6 (13.3)	43.9 (13.3)	43.7 (13.4)	43.4 (13.3)	43.3 (13.2)	0.028
Age, *n* (%)						0.278
18–44	12,366 (51.9)	3384 (50.8)	3199 (51.8)	3093 (52.7)	2690 (52.6)	
45–59	8544 (35.9)	2442 (36.7)	2205 (35.7)	2069 (35.3)	1828 (35.8)	
≥60	2900 (12.2)	837 (12.6)	768 (12.4)	703 (12.0)	592 (11.6)	
Season, *n* (%)						<0.001
Spring	3879 (16.3)	1348 (20.2)	1019 (16.5)	862 (14.7)	650 (12.7)	
Summer	6131 (25.7)	1127 (16.9)	1510 (24.5)	1786 (30.5)	1708 (33.4)	
Fall	6139 (25.8)	1308 (19.6)	1526 (24.7)	1629 (27.8)	1676 (32.8)	
Winter	7661 (32.2)	2880 (43.2)	2117 (34.3)	1588 (27.1)	1076 (21.1)	
Smoking, *n* (%)	4852 (20.9)	1366 (21.2)	1195 (19.9)	1164 (20.3)	1127 (22.5)	0.005
Drinking, *n* (%)	6969 (30.1)	1948 (30.3)	1788 (29.8)	1675 (29.2)	1558 (31.1)	0.186
Systolic blood pressure, mmHg	122.3 (18.4)	122.7 (18.6)	122.3 (18.4)	122.2 (18.4)	122.1 (18.0)	0.335
Diastolic blood pressure, mmHg	71.8 (12.0)	72.2 (12.1)	71.8 (12.0)	71.7 (12.0)	71.5 (11.8)	0.008
Body mass index, kg/m^2^	23.8 (3.4)	23.9 (3.6)	23.9 (3.4)	23.8 (3.3)	23.6 (3.2)	<0.001
Waist circumference, cm	83.0 (10.2)	83.4 (10.8)	83.2 (10.3)	82.9 (10.0)	82.5 (9.5)	<0.001
25(OH)D, ng/mL	18.8 (7.3)	11.9 (3.1)	16.8 (3.6)	20.8 (4.2)	28.0 (6.6)	<0.001
Fasting glucose, mmol/L	5.6 (1.1)	5.7 (1.2)	5.6 (1.1)	5.6 (1.2)	5.6 (1.0)	<0.001
FBI, μU/mL	9.0 (14.3)	9.8 (21.7)	9.0 (13.1)	8.7 (9.6)	8.2 (5.3)	0.001
HOMA-IR	2.4 (5.0)	2.7 (7.4)	2.5 (5.1)	2.3 (3.0)	2.2 (1.7)	0.002
HbA1c, %	5.7 (0.9)	5.7 (0.9)	5.7 (0.9)	5.7 (0.9)	5.6 (0.8)	<0.001
Total cholesterol, mmol/L	5.0 (1.0)	5.0 (1.0)	5.0 (1.0)	5.0 (0.9)	5.0 (1.0)	0.006
LDL-C, mmol/L	3.1 (0.9)	3.0 (0.9)	3.1 (0.9)	3.1 (0.8)	3.1 (0.9)	0.028
HDL-C, mmol/L	1.4 (0.3)	1.3 (0.3)	1.4 (0.3)	1.4 (0.3)	1.4 (0.3)	<0.001
Triglycerides, mmol/L	1.5 (1.4)	1.6 (1.8)	1.5 (1.3)	1.5 (1.2)	1.4 (1.0)	<0.001
Metabolic syndrome, *n* (%)	6231 (27.5)	1907 (30.1)	1680 (28.7)	1485 (26.6)	1159 (23.8)	<0.001
Abdominal obesity, *n* (%)	9307 (40.3)	2651 (41.4)	2501 (41.9)	2248 (39.2)	1907 (38.1)	<0.001
High Triglycerides, *n* (%)	6260 (26.7)	1853 (28.6)	1717 (28.3)	1506 (26.0)	1184 (23.3)	<0.001
Low HDL-C, *n* (%)	4550 (21.9)	1383 (23.5)	1220 (22.6)	1101 (21.7)	846 (19.4)	<0.001
Hypertension, *n* (%)	8055 (34.4)	2284 (35.2)	2079 (34.3)	1988 (34.3)	1704 (33.7)	0.340
Hyperglycemia, *n* (%)	8829 (37.7)	2588 (39.9)	2284 (37.6)	2158 (37.2)	1799 (35.4)	<0.001

Data are shown as mean (SD) or number (%). *p*-values were derived from one-way ANOVA or Pearson’s Chi-squared test. Q1–Q4:. Age-and sex-specific quartiles of serum 25 hydroxyvitamin D.

**Table S1:** Baseline characteristics of participants based on the status of serum 25(OH)D.

Variables	Overall (23,810)	Deficiency (4260)	Insufficiency (10,213)	Sufficiency (9337)
Sex, n (%)				
Male	12,596 (52.9)	1711 (40.2)	5583 (54.7)	5302 (56.8)
Female	11,214 (47.1)	2549 (59.8)	4630 (45.3)	4035 (43.2)
Age, y	43.6 (13.3)	35.8 (11.9)	41.3 (12.7)	49.6 (11.9)
Age, n (%)				
18–44	12,366 (51.9)	3200 (77.0)	6274 (61.4)	2892 (30.6)
45–59	8544 (35.9)	766 (18.4)	3045 (29.8)	4733 (50.1)
≥60	2900 (12.2)	195 (4.6)	897 (8.8)	1808 (19.4)
Season, n (%)				
Spring	3879 (16.3)	755 (17.7)	1846 (18.1)	1278 (13.7)
Summer	6131 (25.7)	663 (15.6)	2593 (25.4)	2875 (30.8)
Fall	6139 (25.8)	651 (15.3)	2357 (23.1)	3131 (33.5)
Winter	7661 (32.2)	2191 (51.4)	3417 (33.5)	2053 (22.0)
Smoking, n (%)	4852 (20.9)	539 (13.2)	1873 (18.8)	2440 (26.5)
Drinking, n (%)	6969 (30.1)	885 (21.7)	3053 (30.8)	3031 (33.0)
25(OH)D, ng/mL	18.8 (7.3)	9.8 (1.6)	16.0 (2.3)	26.1 (5.4)
Abdominal obesity, n (%)	9307 (40.3)	1193 (29.6)	3972 (40.1)	4142 (45.1)
Hypertriglyceridemia, n (%)	6260 (26.7)	795 (19.4)	2780 (27.7)	2685 (28.9)
Low HDL-cholesterol, n (%)	4550 (21.9)	792 (20.4)	2060 (22.8)	1698 (21.7)
Hypertension, n (%)	8055 (34.4)	923 (22.4)	3119 (31.1)	4013 (43.4)
Hyperglycemia, n (%)	8829 (37.7)	1129 (27.4)	3573 (35.6)	4127 (44.5)
Metabolic syndrome, n (%)	6231 (27.5)	739 (18.7)	2629 (27.0)	2863 (32.0)

The authors state that the scientific conclusions are unaffected. This correction was approved by the Academic Editor. The original publication has also been updated.
