# Supplementary material for: Relationship between Circulating 25-Hydroxyvitamin D and Metabolic Syndrome in Chinese Adults: A Large Nationwide Longitudinal Study
Source: Nutrients. 2024 May 14;16(10):1480. doi: 10.3390/nu16101480 (PMC11124364; doi:10.3390/nu16101480)
Supplement: Supplementary file 1 [file nutrients-16-01480-s001.zip › nutrients-2971049-supplementary.pdf]

nutrients MDPI

Table S1. Baseline characteristics of participants based on the status of serum 25(OH)D.

Variables	Overall (23,810)	Deficiency (4260)	Insufficiency (10,213)	Sufficiency (9337)
Sex, n (%)				
Male	12,596 (52.9)	1711 (40.2)	5583 (54.7)	5302 (56.8)
Female	11,214 (47.1)	2549 (59.8)	4630 (45.3)	4035 (43.2)
Age, y	43.6 (13.3)	35.8 (11.9)	41.3 (12.7)	49.6 (11.9)
Age, n (%)				
18–44	12,366 (51.9)	3200 (77.0)	6274 (61.4)	2892 (30.6)
45–59	8544 (35.9)	766 (18.4)	3045 (29.8)	4733 (50.1)
≥60	2900 (12.2)	195 (4.6)	897 (8.8)	1808 (19.4)
Season, n (%)				
Spring	3879 (16.3)	755 (17.7)	1846 (18.1)	1278 (13.7)
Summer	6131 (25.7)	663 (15.6)	2593 (25.4)	2875 (30.8)
Fall	6139 (25.8)	651 (15.3)	2357 (23.1)	3131 (33.5)
Winter	7661 (32.2)	2191 (51.4)	3417 (33.5)	2053 (22.0)
Smoking, n (%)	4852 (20.9)	539 (13.2)	1873 (18.8)	2440 (26.5)
Drinking, n (%)	6969 (30.1)	885 (21.7)	3053 (30.8)	3031 (33.0)
25(OH)D, ng/mL	18.8 (7.3)	9.8 (1.6)	16.0 (2.3)	26.1 (5.4)
Abdominal obesity, n (%)	9307 (40.3)	1193 (29.6)	3972 (40.1)	4142 (45.1)
Hypertriglyceridemia, n (%)	6260 (26.7)	795 (19.4)	2780 (27.7)	2685 (28.9)
Low HDL-cholesterol, n (%)	4550 (21.9)	792 (20.4)	2060 (22.8)	1698 (21.7)
Hypertension, n (%)	8055 (34.4)	923 (22.4)	3119 (31.1)	4013 (43.4)
Hyperglycemia, n (%)	8829 (37.7)	1129 (27.4)	3573 (35.6)	4127 (44.5)
Metabolic syndrome, n (%)	6231 (27.5)	739 (18.7)	2629 (27.0)	2863 (32.0)
